# C-reactive protein and N-terminal prohormone brain natriuretic peptide as biomarkers in acute exacerbations of COPD leading to hospitalizations

**DOI:** 10.1371/journal.pone.0174063

**Published:** 2017-03-22

**Authors:** Yu-wei Roy Chen, Virginia Chen, Zsuzsanna Hollander, Jonathon A. Leipsic, Cameron J. Hague, Mari L. DeMarco, J. Mark FitzGerald, Bruce M. McManus, Raymond T. Ng, Don D. Sin

**Affiliations:** 1 Centre for Heart Lung Innovation, James Hogg Research Centre, St. Paul’s Hospital, Vancouver, British Columbia, Canada; 2 Institute for Heart Lung Health, St. Paul’s Hospital, Vancouver, British Columbia, Canada; 3 PROOF Centre of Excellence, Vancouver, British Columbia, Canada; 4 Department of Radiology, St. Paul’s Hospital, Vancouver, British Columbia, Canada; 5 Department of Pathology and Laboratory Medicine, University of British Columbia, Vancouver, British Columbia, Canada; 6 Division of Respiratory Medicine, Department of Medicine, University of British Columbia, Vancouver, British Columbia, Canada; 7 The Lung Centre, Vancouver General Hospital, Vancouver, British Columbia, Canada; 8 Department of Computer Sciences, University of British Columbia, Vancouver, British Columbia, Canada; National and Kapodistrian University of Athens, GREECE

## Abstract

There are currently no accepted and validated blood tests available for diagnosing acute exacerbations of chronic obstructive pulmonary disease (AECOPD). In this study, we sought to determine the discriminatory power of blood C-reactive protein (CRP) and N-terminal prohormone brain natriuretic peptide (NT-proBNP) in the diagnosis of AECOPD requiring hospitalizations. The study cohort consisted of 468 patients recruited in the COPD Rapid Transition Program who were hospitalized with a primary diagnosis of AECOPD, and 110 stable COPD patients who served as controls. Logistic regression was used to build a classification model to separate AECOPD from convalescent or stable COPD patients. Performance was assessed using an independent validation set of patients who were not included in the discovery set. Serum CRP and whole blood NT-proBNP concentrations were highest at the time of hospitalization and progressively decreased over time. Of the 3 classification models, the one with both CRP and NT-proBNP had the highest AUC in discriminating AECOPD (cross-validated AUC of 0.80). These data were replicated in a validation cohort with an AUC of 0.88. A combination of CRP and NT-proBNP can reasonably discriminate AECOPD requiring hospitalization versus clinical stability and can be used to rapidly diagnose patients requiring hospitalization for AECOPD.

## Introduction

Chronic obstructive pulmonary disease (COPD) is a heterogeneous and debilitating disease that affects 200 million people worldwide and is responsible for 3 million deaths annually [1]. Most of these deaths occur during periods of worsening of symptoms, which are called acute exacerbations of COPD (AECOPD) [1]. Because exacerbations are defined purely based on a health professionals’ interpretation of patient symptoms, the accuracy of the current definition of AECOPD is uncertain. This may in part explain the heterogeneity of clinical presentation and outcomes of these events and the variable response to therapy [2–4]. There is a pressing need to objectify these events to enable more accurate endotyping of these events and to ensure prompt implementation of appropriate therapy.

Most AECOPD events are precipitated by an acute respiratory tract infection, usually viral in etiology [5]. Other causes of AECOPD include: poor adherence to medication, acute cardiovascular events, pulmonary embolism, and bacterial infections including those that are caused by gram negative bacilli which carry lipopolysaccharide (LPS) in their walls [6–9]. C-reactive protein (CRP) is an acute-phase systemic inflammatory biomarker that is known to be associated with COPD exacerbations [10, 11]. Circulating CRP concentrations are generally greater in COPD patients compared to healthy controls [12, 13], and can rise to even higher concentrations during AECOPD [11]. During clinical stability, CRP is associated with all-cause, cardiovascular, and cancer mortality [14].

Brain natriuretic peptide (BNP) has been used clinically to screen and diagnose acute decompensated heart failure [15]. The main stimulus for BNP release is mechanical stress in the cardiomyocytes related to volume overload [16]. In the context of COPD, BNP concentrations have been shown to be elevated compared to healthy controls [17] and in AECOPD versus stable COPD patients [18]. Amino-terminus of the prohormone BNP (NT-proBNP) is the inactive fragment that is released in conjunction with BNP in a 1:1 ratio [19]. NT-proBNP has been investigated as a possible biomarker in AECOPD with or without left ventricular dysfunction [20], in AECOPD with respiratory failure [21], and in AECOPD with ischemic heart disease [22].

To our knowledge, CRP and NT-proBNP have not been studied concurrently in the same patient over the full time course of AECOPD requiring hospitalization. As inflammatory events associated with exacerbation can impact both the pulmonary and cardiac systems, our aim was to first investigate the temporal relationship between CRP and NT-proBNP during AECOPD. We hypothesized that CRP and NT-proBNP concentrations are elevated in AECOPD and can be used to distinguish between exacerbating versus stable COPD patients. Our second aim was to develop discriminatory models based on CRP and NT-proBNP singly and in combination, and to replicate these models. Part of this study has been presented in abstract form previously [23, 24].

## Methods

### Study subjects

**Fig 1:**
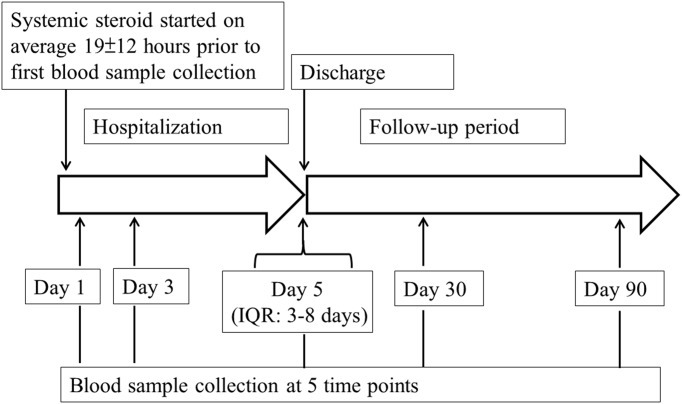
Timeline of blood collection and systemic steroids administration. The figure displays blood sample collection time point during hospitalization to follow-up for each patient visit. Samples were collected at day 1 of hospitalization, day 3, discharge, day 30 and day 90 post-hospitalization. Systemic corticosteroids were administered on average 19±12 hours prior to the first blood sample collection after hospital admission. The day of discharge is variable, with a median of 5 days and interquartile range (IQR) of 3–8 days.

This observational study includes patients recruited into the COPD Rapid Transition Program. The cohort consisted of a total 468 AECOPD patients who were hospitalized at St Paul’s Hospital or Vancouver General Hospital in Vancouver, British Columbia. All patients included in the analysis had a confirmed primary diagnosis of AECOPD as deemed by general internists or pulmonologists who cared for these patients. Two independent physicians who were not involved in the care of the patients subsequently reviewed and validated the diagnoses based on chart review. If the primary diagnoses assessed by the two reviewers did not agree, the patients were excluded from our analysis. Patients with known comorbidities, such as kidney disease were also excluded from the analysis. All the patients included in this analysis received standard anti-exacerbation treatment during their hospitalization, including short-acting bronchodilators, prednisone and antibiotics as necessary (see Fig 1). In addition to the hospitalized AECOPD cohort, 110 stable COPD patients (different than the patients in the AECOPD cohort) were recruited from the St. Paul’s Hospital COPD clinic, who had been free of AECOPD for at least 8 weeks and they served as non-exacerbating COPD controls. The entire cohort was then divided into 1) a discovery set consisting of 421 AECOPD and 76 stable COPD patients (recruitment between July 2012 and early April 2015) and 2) a validation set consisting of 47 AECOPD and 34 stable COPD patients (recruitment between late April 2015 to May 2016). The study is registered on ClinicalTrials.gov website with Identifier: NCT02050022 (registered January 28, 2014). The study was approved by the University of British Columbia Clinical Research Ethics Board (certificate number H11-00786). Written informed consent was provided by each participant in accordance with the Ethics Board.

### Specimens and measurement technique

Following informed consent, blood samples were collected from patients in PAXgene^®^, EDTA, and serum tubes on day 1 and 3 of hospitalization, at discharge, and on day 30 and day 90 post-admission date (Fig 1). Blood components were processed as per standardized protocol and stored at -80°C until analysis.

Serum CRP was measured via a high-sensitivity assay on the Advia^®^ 1800 Chemistry System (Siemens Healthcare GmbH, Erlangen, Germany) in the Clinical Laboratory of St Paul’s Hospital (Department of Pathology and Laboratory Medicine, Vancouver, BC, Canada) following standard operating procedures. NT-proBNP was measured from EDTA whole blood specimens on the RAMP^®^ 200 (Response Biomedical Corp, Vancouver, BC, Canada), which had a measurement range of 18 to 35,000 ng/L.

Baseline lung function measurements were performed by spirometry after bronchodilator administration at the time of convalescence (i.e. at day 30 or day 90) for AECOPD patients, and at COPD clinic visits for stable COPD control patients. The presence of pulmonary edema was assessed on chest X-ray images by an experienced chest radiologist who was blinded to the characteristics of the study participants.

### Statistical analysis

Continuous variables that were not normally distributed were transformed using a natural logarithm prior to a Student’s t-test analysis. Categorical dichotomous variables were compared using a chi-square test. P-values of less than 0.05 (on a two-tailed t-test) were considered statistically significant. The association between CRP and NT-proBNP concentrations was determined by Pearson’s correlation. The multiple linear regression analysis was used to model the length of hospitalization versus the two biomarkers, and was adjusted for age, sex and current smoking status. Cox proportional hazards model was used for either CRP or NT-proBNP concentration at exacerbation onset to predict the risk of death. Likelihood ratio test was used in calculating p-values.

Receiver-operating characteristic (ROC) curves were generated based on logistic regression models for diagnosing AECOPD (day 1) from convalescent (day 30 or 90) and stable COPD samples. We compared the area under the ROC curve (AUC) of 3 models: 1) NT-proBNP, 2) CRP, and 3) a model built on both CRP and NT-proBNP, with discovery probabilities generated using leave-one-out cross-validation (LOOCV). The AUCs were compared across models using Hanley and McNeil method [25]. In the LOOCV model, the algorithm cycled through the complete dataset, and a sample was excluded systematically (total n–1) with each iteration. Then the models were subsequently computed based on the total n–1 samples, and tested on the one sample that was left out. This method provided a way of estimating performance and minimized over-fitting of data to the discovery set as previously recommended [26]. The “optimal” cut-off value for the biomarkers was determined based on models that gave 90% specificity. All statistical analysis was performed using R.

Additional details on the study cohort, measurement techniques, and statistical analysis are provided in S1 File.

## Results

Demographic data and lung function measurements of the discovery set are shown in Table 1. There were no significant differences in the baseline characteristics between patients with AECOPD and stable patients except for smoking status, and the use of inhaled corticosteroids, and prednisone. Additional data on the validation set are in Table A of the S1 File.

**Table 1:** Patient characteristics of the discovery set.

		AECOPD	COPD Stable Controls	P-value
Age (years)		66.9±11.8	68.2±10.8	0.190
Sex (Male %)		60.0%	66.6%	0.155
BMI (kg/m^2^)		26.4±7.3	26.3±6.7	0.887
Ethnicity (Caucasian %)		85.1%	82.8%	0.540
Smoking	Current (%)	62.6%	50.9%	0.013
	Former (%)	30.8%	43.8%	
	Unknown (%)	0.5%	1.8%	
	Never (%)	6.7%	5.3%	
Smoking Duration (pack-years)		54.2±41.8	53.0±34.6	0.729
FEV1% predicted		54.6±22.6	54.7±20.1	0.983
FVC % predicted		77.8±23.7	77.5±18.8	0.900
FEV1 / FVC (%)		55.7±15.5	56.1±22.0	0.898
GOLD Stages	I	16.5%	13.6%	0.833
	II	37.6%	42.6%	
	III	32.9%	32.4%	
	IV	12.9%	11.4%	
Congestive Heart Failure		18.6%	17.3%	0.722
Coronary Artery Disease		25.8%	27.4%	0.758
Hypertension		53.6%	50.0%	0.466
Pulmonary Edema*	None (%)	84.5%	81.6%	0.778
	Mild (%)	10.3%	13.2%	
	Moderate-Severe (%)	5.2%	5.2%	
Inhaled Steroids (%)		37.8%	75.9%	<0.0001
Prednisone (%)		6.2%	0.47%	0.001
D-Dimer (μg/L FEU)		499[304–945]	364[220–740]	0.205
Cardiac Troponin-I (μg/L)		0.00[0.0–0.0]	0.00[0.0–0.0]	0.109
Serum Creatinine (μmol/L)		83[58–98]	89[61–105]	0.123

Continuous variables are presented as mean ± SD or median[IQR] if not normally distributed. Comparisons made via t-test after natural-log transformation. Dichotomous variable are presented as counts (% total). Comparisons are made via chi-square test. Cardiac troponin-I and D-Dimer concentrations measured from AECOPD day 1 samples. Lung Function measurements taken from post-bronchodilator stable/convalescent samples (i.e. at Day 30 or Day 90). Abbreviations: BMI, body mass index, FEU = Fibrinogen equivalent unit, GOLD = Global Initiative for Chronic Obstructive Lung Disease, IQR = interquartile range, and SD = standard deviation. *based on radiologist’s interpretation of chest radiograph taken at the time of hospital admission for AECOPD patients and at a time of clinical stability for control patients.

### Time course

**Fig 2:**
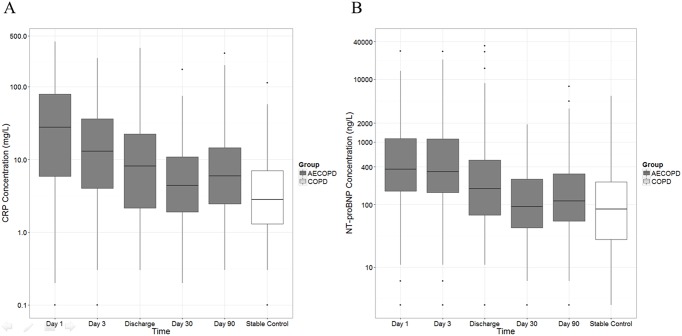
CRP and NT-proBNP time course box-plots. **A)** CRP concentrations of the discovery set at five time-points for AECOPD patients and as well as stable COPD controls. The data are expressed as Tukey box-plots, in which the box represents the 25th, the median, and the 75th percentile. The whiskers extend to 1.5 times of the interquartile range on either side of the box, and the outliers plotted separately. The y-axis is displayed on a natural-log scale. **B)** NT-proBNP concentrations of the discovery set represented similarly to **A**.

Box-plots of CRP in the discovery set are shown in Fig 2A. Median CRP concentrations were highest on day 1 at 27.7[5.9–78.9] mg/L (median[IQR]), 8.5[2.2–22.4] mg/L at discharge, and 5.8[2.1–14.0] mg/L at day 90 (p<0.001 for day 1 versus discharge and day 90, respectively). The day 1 CRP concentrations were significantly higher than that of stable controls (2.8[1.3–7.1] mg/L; p<0.001). There was no significant difference between day 30 CRP compared to the stable group, however, there was a significant difference between day 90 versus stable (p = 0.021).

Box-plots of NT-proBNP, which summarize data from the discovery set, are displayed in Fig 2B. NT-proBNP median concentrations were highest on day 1 with 367[168–1189] ng/L and significantly decreased over time (187[69–544] ng/L at discharge and 115[46–309] ng/L at day 90; p = 0.043 and p<0.001, respectively). The day 1 concentration was also significantly greater than the stable controls (85[28–230] ng/L; p<0.001). There was no significant difference between day 30 and day 90 versus the stable group.

### Correlations of CRP and NT-proBNP

The relationship between CRP and NT-proBNP are shown using a scatter plot in S1 Fig. There was a modest positive linear association between the two biomarkers with a correlation coefficient r = 0.253 (p<0.001).

### Length of stay and mortality

**Fig 3:**
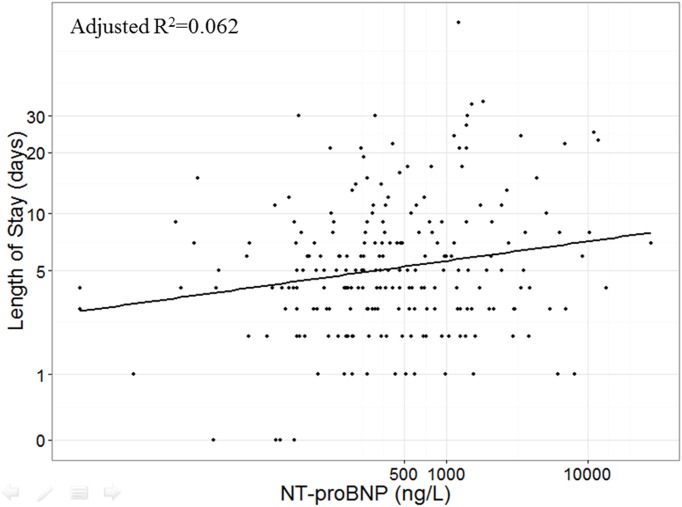
Length of hospital stay versus NT-proBNP concentration. NT-proBNP and length of stay are both plotted on natural-log scale. The linear relationship is significant (p = 0.042) from a multiple linear regression analysis. The axes are on a natural-log scale. The model has an adjusted R-squared value of 0.062 based on a sample size of 222 patients from the discovery set. The model was adjusted for age, gender and smoking status.

**Fig 4:**
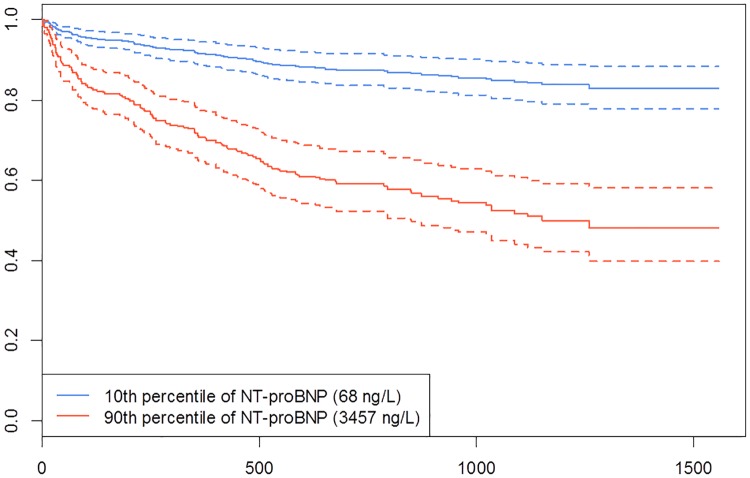
NT-proBNP Cox proportional hazards survival curve. The figure shows the concentration of NT-proBNP at the time of hospitalization in predicting all-cause mortality. The x-axis represents number of days post-hospitalization and the y-axis represents the proportion of survivors. The red survival curve followed patients with high NT-proBNP concentrations (90^th^ percentile) whereas the blue curve followed patients with low NT-proBNP concentrations (10^th^ percentile). The curves were displayed with 95% confidence intervals as dotted lines. There was a significant hazard ratio of 1.27 [CI: 1.18–1.38] risk associated with the doubling of NT-proBNP concentration (p-value < 0.0001).

Table 2 summarizes the variables from the multiple linear regression model. Only NT-proBNP concentrations taken at day 1 of the hospitalization was statistically significantly related to total length of stay with a p-value = 0.042 (Fig 3). Fig 4 shows the Cox proportional hazards model of NT-proBNP concentrations at exacerbating onset (i.e. first sample during hospitalization) in predicting all-cause mortality. There was a significant hazard ratio of 1.27 [CI: 1.18–1.38] risk associated with the doubling of NT-proBNP concentration (p-value < 0.0001). In contrast, CRP concentrations at exacerbation onset were not significant in predicting death.

**Table 2:** Multiple linear regression analysis in modeling length of hospitalization.

Variable	β estimate	Standard Error	95% CI for β	P-value
CRP (mg/L)	0.040364	0.028066	-0.014645–0.095373	0.152
NT-proBNP (ng/L)	0.063431	0.031033	0.002608–0.124254	0.042
Age	0.008168	0.004445	-0.000545–0.016880	0.068
Sex (Male)	0.029916	0.094984	-0.156250–0.216081	0.753
Current Smoker (Yes)	-0.116929	0.104867	-0.322466–0.088607	0.266

The length of stay, CRP, and NT-proBNP values are natural-log transformed prior to regression analysis (n = 222). The concentrations were based on the first AECOPD samples.

### Classification

**Fig 5:**
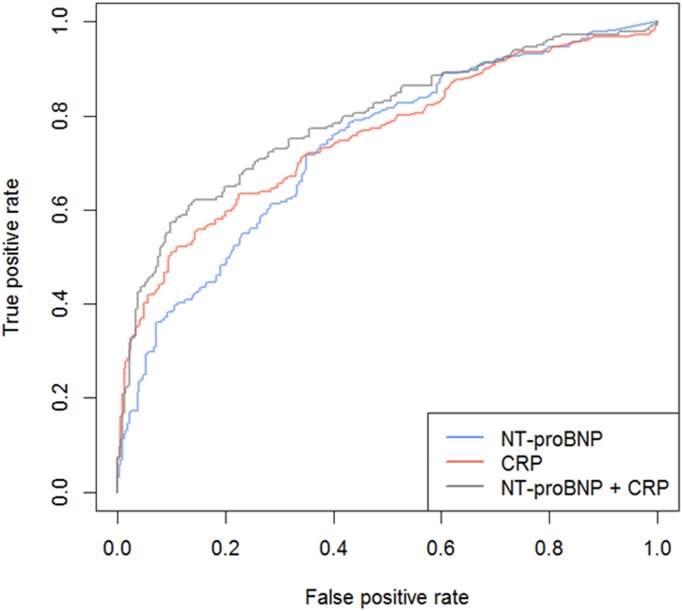
ROC curves of the 3 models from the discovery set. ROC curve for 1) CRP, 2) NT-proBNP, and 3) CRP + NT-proBNP. The ROC curve is used in discriminating patients with AECOPD. Abbreviations: CRP = C-reactive protein, and NT-proBNP = N-terminal of the prohormone brain natriuretic peptide.

ROC curves of the discovery set are displayed in Fig 5. The AUC for CRP alone was 0.77, NT-proBNP alone was 0.75, and combined, the LOOCV model had a cross-validated AUC of 0.80. The corresponding performances for various cut-off thresholds are shown in Table 3. AUC comparisons between CRP + NT-proBNP model versus CRP and NT-proBNP alone models produced p-values of 0.089 and 0.044, respectively. The optimal cut-offs for NT-proBNP alone and CRP alone, based on the discovery samples, were 590 ng/L and 26 mg/L, respectively. The linear predictor for the logistic regression model built using both CRP and NT-proBNP was:

Linear predictor= −0.15+0.029*CRP+0.00055*NTproBNP

**Table 3:** ROC curve performances of the 4 models in the discovery set.

	AUC	AUC 95% CI	Sensitivity	Specificity	Cut-off value	P-value (CRP + NT-proBNP vs each single-marker models)
CRP (mg/L)	0.77	0.74–0.77	54%	90%	26	0.089
NT-proBNP (ng/L)	0.75	0.71–0.75	39%	90%	590	0.044
CRP + NT-proBNP	0.80	0.77–0.81	58%	90%	0.395*	N/A
D-Dimer (μg/L FEU)	0.56	0.54–0.57	11%	94%	1737	<0.0001

Performance metrics in the ROC curve analysis. AUC, sensitivity, and specificity are based on discrimination between AECOPD versus stable COPD samples. The p-values were computed for AUC comparisons between the CRP + NT-proBNP model versus each single marker respectively. The abbreviations: AUC = Area under the curve, CI = confidence interval, CRP = C-reactive protein, N/A = Not applicable, NT-proBNP = N-terminal prohormone of brain natriuretic peptide, and FEU = Fibrinogen equivalent unit.

* There are numerous combinations of CRP and NT-proBNP values that could exceed the cut-off of 0.395 as it is based on the linear predictor from the logistic regression model.

Coefficients for both CRP and NT-proBNP were significant (p<0.001). The optimal cut-off for this combinatorial model was 0.395.

### Replication in the test subjects

Box-plots of CRP and NT-proBNP in the validation set are shown in S2A and S2B Fig respectively. Both biomarkers replicated the trend observed in the discovery set. The three discriminative models were applied in the validation set and the ROC curve performances are shown in S3 Fig. The models replicated with an AUC of 0.86 for CRP alone, 0.79 for NT-proBNP alone, and 0.88 for the model combining CRP and NT-proBNP. The corresponding performance metrics for all 3 models are shown in Table 4.

**Table 4:** ROC curve performances of the 4 models in the validation set.

	AUC	AUC 95% CI	Sensitivity	Specificity	P-value (CRP + NT-proBNP vs each single-marker models)
CRP (mg/L)	0.86	0.82–0.90	55%	92%	0.318
NT-proBNP (ng/L)	0.79	0.74–0.83	48%	95%	0.065
CRP + NT-proBNP	0.88	0.85–0.92	71%	90%	N/A
D-Dimer (μg/L FEU)	0.65	0.59–0.71	22%	91%	0.001

Performance metrics in the ROC curve analysis for the validation set. AUC, sensitivity, and specificity are based on discrimination between AECOPD versus stable COPD samples. The p-values were computed for AUC comparisons between the CRP + NT-proBNP model versus each single marker respectively. The abbreviations: AUC = Area under the curve, CI = confidence interval, CRP = C-reactive protein, N/A = Not applicable, NT-proBNP = N-terminal prohormone of brain natriuretic peptide, and FEU = Fibrinogen equivalent unit.

## Discussion

In this study, we noted the following key findings: firstly, circulating blood CRP and NT-proBNP were both elevated during AECOPDs requiring hospitalization and decreased with treatment during the recovery phase of the hospitalization; secondly, the two biomarkers were, however, weakly correlated and NT-proBNP but not CRP concentrations were significantly associated with total mortality and length of hospitalizations, suggesting that they may reflect distinct pathways and may correspond to different endotypes of AECOPD; thirdly, using the LOOCV method, a combination of CRP and NT-proBNP discriminated with reasonable AUCs patients experiencing acute hospitalization for their AECOPD from those who were stable; and lastly, our models were replicated in a validation set of COPD patients with reproducible AUCs that were comparable to the discovery set, providing support for the use of these two biomarkers in combination to diagnose AECOPD.

Our finding of elevated blood CRP during exacerbation is consistent with published literature [10, 11, 13, 27, 28]. Given the role of CRP as an acute-phase reactant, elevated concentrations at day 1 likely reflect the impact of respiratory infection on the host inflammatory responses in the systemic circulation. With resolution of the inflammatory process, there is a gradual decay in CRP over time reaching a nadir at day 30 to 90 post-hospitalization (Fig 2A). During this period, there is approximately a 4.5-fold difference in CRP from day 1 of hospitalization to recovery. The exact mechanism for this observation is not fully known. Corticosteroids, which are often used in AECOPD treatment, have been shown to reduce CRP concentrations [29]. The CRP reduction may also reflect removal (or resolution) of the inciting event (e.g. viral or bacterial infection). Interestingly, CRP concentrations at day 90 were significantly higher than those of stable COPD controls. One possible explanation could be related to the cessation of systemic corticosteroids, which may have led to a small “rebound” effect on CRP [13, 29].

The rise of NT-proBNP during AECOPD hospitalizations is also consistent with published literature [21]. The box-plots in Fig 2B showed a significant decrease in blood NT-proBNP concentrations from day 1 to discharge (approximately a 3-fold change). BNP concentrations have been reported to be elevated in patients with renal failure, independent of heart failure [30]. Part of the clearance method is through passive excretion, in which the glomerular filtration rate is inversely related to BNP/NT-proBNP concentrations [31]. Calzetta et al. recently reviewed the teleological role of BNP/NT-proBNP [32]. The receptor for BNP, natriuretic peptide receptor A (NPR-A), has been identified in various respiratory cells, such as type II alveolar cells and endothelial cells of pulmonary blood vessels. This suggests that BNP may modulate the respiratory system, in addition to its traditional role as a heart failure biomarker. Although the actual mechanisms are yet to be elucidated, the authors suggested BNP/NT-proBNP as treatment monitoring biomarkers for the management of patients with AECOPD.

In our cohort, the elevated values seen on Fig 2B were unlikely due to renal impairment, as patients with severe kidney disease were excluded from our analysis. Furthermore, the serum creatinine concentrations in our cohort were within the appropriate reference intervals (see Table 1). Venous thromboembolism (VTE), which can induce AECOPD, can also elevate NT-proBNP concentrations. To investigate this possibility, we measured blood D-dimers, which are biomarkers of pulmonary embolism, during AECOPD events. However, D-dimer concentrations during AECOPDs were no different than those during stability, suggesting that the elevated NT-proBNP during AECOPDs were not caused by occult VTE.

A significant portion of our patients had mildly elevated NT-proBNP concentrations (i.e. 367–1189 ng/L) during day 1 and 3, which were not in the range of overt left-sided congestive heart failure. Findings based on review of chest X-ray would suggest the prevalence of overt pulmonary (venous) congestion was low in our cohort. The mild elevation in NT-proBNP could have arisen from mild right or left cardiac dysfunction related to AECOPD, which may go undetected on X-ray imaging. A previous study has shown that a small proportion of AECOPD patients with high BNP concentrations have diastolic and systolic dysfunction [18]. Study by Segreti et al., in which the patients were either given salbutamol or indacaterol, also found mild elevations of BNP levels at AECOPD onset for both arms of the trial. They reported a mean concentration of 381 and 206 pg/mL (salbutamol and indacaterol, respectively) at AECOPD onset, which decreased by day 5 [33]. Although there is no direct conversion factor between BNP and NT-proBNP assays, a BNP cut-off value of 400 ng/L would indicate severe congestive heart failure, whereas, a concentration lower than 400 ng/L would indicate mild to moderate heart failure. Similar to our cohort, the BNP concentrations reported by Segreti et al. would be considered mildly elevated as they fell between the ranges of 100–400 ng/L. Another study reported elevations in both NT-proBNP and troponin-T concentrations in AECOPD patients admitted to the intensive care unit (ICU) with left ventricular dysfunction [20]. It has been reported that the prevalence of COPD in chronic heart failure patients is approximately 20–26% [34, 35]. In our cohort, the prevalence of unequivocal (concomitant) heart failure (defined as NT-proBNP of 1,000 ng/L or greater) was 5.2%. These patients had NT-proBNP concentrations above the 75^th^ percentile of the Fig 2B box-plot (3603[1092–8235] ng/L) during exacerbation onset. However, there was little evidence that these patients experienced acute cardiac injury, as indicated by normal cardiac troponin-I concentrations, and no radiographic evidence of left ventricular failure from chest X-ray (see Table 1).

While it was beyond the purview of this study to ascertain the mechanisms responsible for these observations, we believe that the most plausible explanation was that AECOPD patients developed mild cardiac distress secondary to the exacerbations. It is well known that exacerbations in COPD patients can lead to dynamic lung hyperinflation [36], which in turn, could stress the cardiomyocytes and suborn their release of NT-proBNP. Another possibility is that lung inflammation during AECOPD may spill over into the systemic circulation, causing myocardial stress and cardiac dysfunction, which in turn may lead to elevated NT-proBNP concentrations. In a murine model, acute instillation of LPS directly into lungs induced a transient state of cardiac dysfunction (with reduced cardiac output) [9]. Interestingly, inhibition of interleukin-6 (IL-6) prevented cardiac dysfunction in this model, suggesting that lung and systemic inflammation plays a critical role in the pathogenesis of myocardial dysfunction related to acute lung injury. While there is a scarcity of human data, it is conceivable that the inflammatory response during AECOPD may be an important driver of (subclinical) myocardial dysfunction (leading to mild elevations in NT-proBNP).

### Classification

To our knowledge, this is the first study to examine the combined utility of CRP and NT-proBNP in differentiating patients who were experiencing acute hospitalization for COPD from stable patients. The performance characteristics we observed from a logistic regression model suggest that the combinatorial usage of the two biomarkers agrees with the physician diagnosis approximately 80% of the time. As suggested in previous studies, an AUC of 0.80 is considered “good” in terms of discriminatory performance [11, 26, 37]. Our findings are consistent with previously published smaller studies, which have evaluated CRP either alone or in conjunction with other biomarkers in the diagnosis of AECOPD [11, 27, 38]. A study by Gumus et al. examined 43 AECOPD patients admitted to the hospital and reported an AUC of 0.695 using CRP from day 1 and 7 [27]. Another study, by Helmy et al., reported using CRP with IL-6 to diagnose AECOPD patients admitted to ICU, and in predicting 28-day mortality with an AUC of 0.851 [38]. Finally, Hurst et al. reported an AUC of 0.73 for CRP alone, and with the addition of a major symptom, improved the AUC to 0.88 [11]. In our study, we obtained an AUC of 0.77 for CRP alone, and an AUC of 0.80 for the combined CRP and NT-proBNP model even without the addition of symptoms, and in the validation set, the AUC was 0.88 using a very conservative (but more valid and reproducible) statistical model, LOOCV, than the traditional ROC-AUC analysis. Although the LOOCV model of CRP + NT-proBNP was not statistically better compared to the model containing CRP alone (p = 0.089), we believe that the combination approach is more promising than CRP alone as a biomarker of AECOPD for several reasons. First, if we tune the biomarker to have a specificity cut-off of 90%, CRP + NT-proBNP combination has a superior sensitivity of 58% compared with that of CRP or NT-proBNP alone, which has a sensitivity of 54% and 39%, respectively (see Table 3). More importantly, the combination marker was significantly associated with important clinical outcomes such as mortality and length of stay in hospital; whereas CRP alone was not. This suggests that the combination biomarker can potentially identify “sicker” AECOPD patients and enable more aggressive management approaches for these patients. Alternative explanation is that the current standard therapy of corticosteroids and antibiotics may address the infectious and inflammatory component of AECOPD but not the cardiac dysfunction/pulmonary congestion components. Future studies will be required to optimize the use of these biomarkers in the clinics.

The strength of this study is in the time course of CRP and NT-proBNP blood concentrations, spanning a total duration of approximately 90 days with a large sample size of 468 well-characterized AECOPD patients. Although our time course contains five time-points similar to studies published from other groups [22, 39], our design covered a wider duration of time-points beyond 8 weeks. Our data showed that these two biomarkers are modifiable and respond well to the progression of AECOPD treatment, and therefore, they can be used potentially as end-points in future AECOPD biomarker studies. We determined that the combinatorial model provided substantial discriminatory power to differentiate exacerbation versus stable COPD patients, and this was replicated in a validation set. The combinatorial method is not a new concept, and has been utilized in other diseases [40]. Here in our study, we propose the use of a common systemic inflammatory biomarker along with a cardiac stress biomarker as the starting point to diagnose COPD exacerbations. This combinatorial approach could potentially be expanded to more biomarkers in the future, and then the new additions could be assessed on whether they add any significant incremental value.

Our study has a few limitations. First, we lacked “true” baseline values of the two biomarkers prior to exacerbation for our cohort. This was due to the nature of our recruitment design, in which we enrolled patients when they experienced a full-blown COPD exacerbation that required hospitalization. We used convalescent samples from day 30 or day 90 as an alternative representation of baseline for our patients. Second, we made the assumption that the day 1 samples from our cohort were taken at the peak of exacerbation. However, this may not have been the case in all patients and it was unclear how long the patients waited prior to seeking medical attention. This could have contributed to the overall variability in our biomarker measurements. Third, the ROC optimal cut-off for the combinatorial models cannot be easily interpreted as concentrations. One would need to compute the biomarker concentrations into a logistic equation and solve for the response variable to determine whether it is higher or lower than the cut-off. Although the calculations can be done computationally in a program (e.g. R), it is not straightforward. Fourth, given that the Rapid Transition cohort is an ongoing prospective study, we acknowledge that the discovery/validation split ratio may not be statistically optimal. Ideally, a 50:50 split in the ratio between discovery and validation cohort sizes would have enhanced the statistical power of the study. Fifth, we used X-ray images to evaluate for heart failure. Echocardiographic assessment would have provided confirmatory measurements of systolic and diastolic function. Sixth, we used clinical impression as the gold standard to assess the performance of the biomarkers. Clinical assessment is variable and fallible. Thus, it is not the ideal gold standard for evaluating biomarkers for AECOPD, and not surprising that we did not achieve AUCs that were 0.90 or greater. Notwithstanding, the concentrations of our two biomarkers (and in particular NT-proBNP) related to key outcome measures including length of hospitalization and mortality, highlighting the potential value-addedness of these biomarkers in the diagnosis of AECOPD.

In summary, both CRP and NT-proBNP concentrations are significantly elevated during AECOPD and decreased with treatment and in recovery. A combinatorial approach could separate patients who were experiencing AECOPD that required hospitalization from stable patients. These two proteins are promising biomarkers for diagnosing and tracking AECOPD progression, and provide the foundation for future biomarker studies.

## Supporting information

S1 Fig CRP and NT-proBNP correlation scatter plot. The scatter plot shows 400 pairs of CRP and NT-proBNP results based on the first sample collected during AECOPD hospitalization. The concentrations are plotted on logarithmic scales on both axes. The linear regression line is plotted with shaded region being the 95% confidence intervals.
(TIF)

S2 Fig CRP and NT-proBNP time course box-plots. **A)** CRP concentrations of the validation set at five time-points for AECOPD patients and as well as stable COPD controls. The data are expressed as Tukey box-plots, in which the box represents the 25th, the median, and the 75th percentile. The whiskers extend to 1.5 times of the interquartile range on either side of the box, and the outliers plotted separately. The y-axis is displayed on a natural-log scale. **B)** NT-proBNP concentrations of the validation set represented similarly to **A**.
(TIF)

S3 Fig ROC curves of the 3 models from the validation set. ROC curve for 1) CRP, 2) NT-proBNP, and 3) CRP + NT-proBNP. The ROC curve is used in discriminating patients with AECOPD. Abbreviations: CRP = C-reactive protein, and NT-proBNP = N-terminal of the prohormone brain natriuretic peptide.
(TIF)

S1 File Supporting information methods and tables. The file contains a methods section providing further information on study subjects, specimen collection and measurement technique, and statistical analysis. Table A within the S1 file displays a table of patient characteristics of the validation set.
(DOCX)
