# Supplementary figures and images for: C-reactive protein and N-terminal prohormone brain natriuretic peptide as biomarkers in acute exacerbations of COPD leading to hospitalizations
Source: PLoS One. 2017 Mar 22;12(3):e0174063. doi: 10.1371/journal.pone.0174063 (PMC5362097; doi:10.1371/journal.pone.0174063)

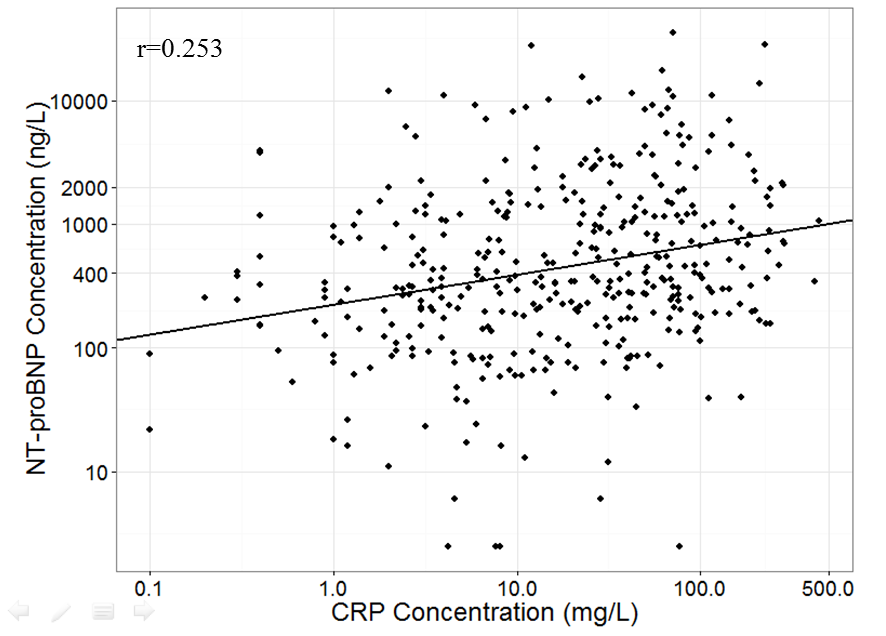

Supplement: S1 Fig — The scatter plot shows 400 pairs of CRP and NT-proBNP results based on the first sample collected during AECOPD hospitalization. The concentrations are plotted on logarithmic scales on both axes. The linear regression line is plotted with shaded region being the 95% confidence intervals. (TIF) [file pone.0174063.s001.tif]

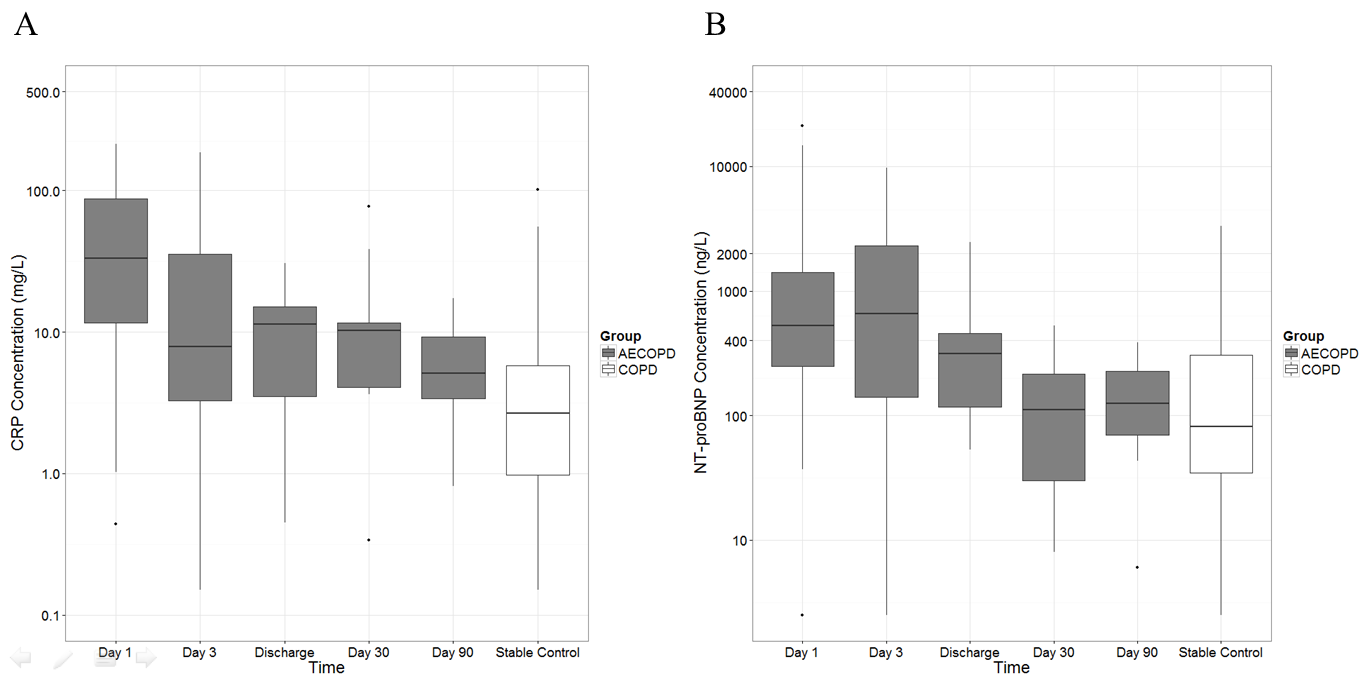

Supplement: S2 Fig — A) CRP concentrations of the validation set at five time-points for AECOPD patients and as well as stable COPD controls. The data are expressed as Tukey box-plots, in which the box represents the 25th, the median, and the 75th percentile. The whiskers extend to 1.5 times of the interquartile range on either side of the box, and the outliers plotted separately. The y-axis is displayed on a natural-log scale. B) NT-proBNP concentrations of the validation set represented similarly to A. (TIF) [file pone.0174063.s002.tif]

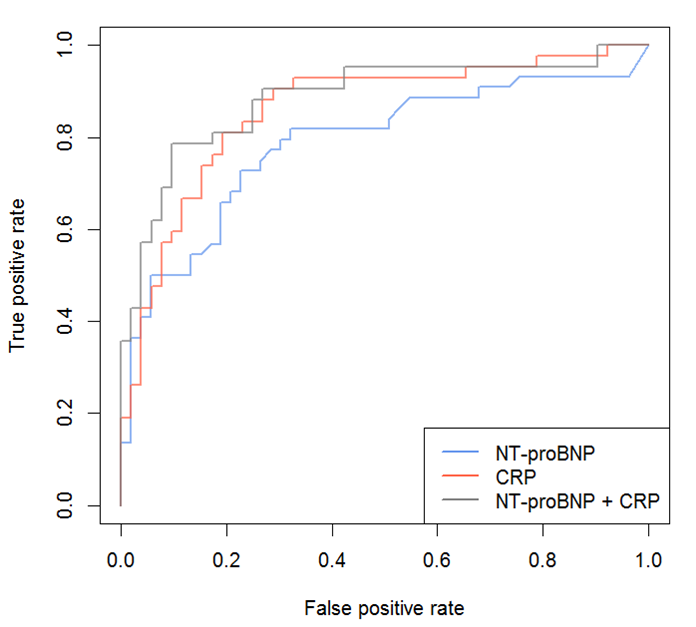

Supplement: S3 Fig — ROC curve for 1) CRP, 2) NT-proBNP, and 3) CRP + NT-proBNP. The ROC curve is used in discriminating patients with AECOPD. Abbreviations: CRP = C-reactive protein, and NT-proBNP = N-terminal of the prohormone brain natriuretic peptide. (TIF) [file pone.0174063.s003.tif]
