# Supplementary material for: C-reactive protein and N-terminal prohormone brain natriuretic peptide as biomarkers in acute exacerbations of COPD leading to hospitalizations
Source: PLoS One. 2017 Mar 22;12(3):e0174063. doi: 10.1371/journal.pone.0174063 (PMC5362097; doi:10.1371/journal.pone.0174063)
Supplement: S1 File — The file contains a methods section providing further information on study subjects, specimen collection and measurement technique, and statistical analysis. Table A within the S1 file displays a table of patient characteristics of the validation set. (DOCX) [file pone.0174063.s004.docx]

C-reactive Protein and N-Terminal Prohormone Brain Natriuretic Peptide as Biomarkers in Acute Exacerbations of COPD Leading to Hospitalizations

Yu-Wei Roy Chen^1^, Virginia Chen^1,2,3^, Zsuzsanna Hollander^1,2,3^, Jonathon A. Leipsic^4^, Cameron J. Hague^4^, Mari L. DeMarco^5^, J. M. FitzGerald^6,7^, Bruce M. McManus^1,2,3,5^, Raymond T. Ng^3,8^, Don D. Sin^1,2,6*^

^1^ Centre for Heart Lung Innovation, James Hogg Research Centre, St. Paul’s Hospital, Vancouver, British Columbia, Canada

^2^ Institute for Heart Lung Health, St. Paul’s Hospital, Vancouver, British Columbia, Canada

^3^ PROOF Centre of Excellence, Vancouver, British Columbia, Canada

^4^ Department of Radiology, St. Paul’s Hospital, Vancouver, British Columbia, Canada

^5^ Department of Pathology and Laboratory Medicine, University of British Columbia, Vancouver, British Columbia, Canada

^6^ Division of Respiratory Medicine, Department of Medicine, University of British Columbia, Vancouver, British Columbia, Canada

^7^ The Lung Centre, Vancouver General Hospital, Vancouver, British Columbia, Canada

^8^ Department of Computer Sciences, University of British Columbia, Vancouver, British Columbia, Canada

* Corresponding author

Email: Don.Sin@hli.ubc.ca (DS)

**Supporting Information Methods**

**Study subjects**

This observational study includes patients recruited into the COPD Rapid Transition Program between July 2012 and December 2016. At the time of writing, the cohort consisted of a total 468 AECOPD patients who were hospitalized at St. Paul’s Hospital or Vancouver General Hospital in Vancouver, British Columbia. All patients included in the analysis have a confirmed primary diagnosis of AECOPD as deemed by general internists or pulmonologists who cared for these patients during their hospitalization. Two independent physicians who were not involved in the care of the patients subsequently reviewed and validated the diagnoses based on chart review. If the primary diagnoses assessed by the two reviewers did not agree, the patients were then excluded from our analysis. Patients with known comorbidities, such as kidney disease were also excluded from the analysis. All the patients included in this analysis received standard anti-exacerbation treatment during their hospitalization, including short-acting bronchodilators, prednisone and antibiotics as necessary (see Fig 1). Upon discharge, a team of healthcare professionals including research coordinators and nurses followed the patients. During the follow up, data on all-cause mortality post-hospitalization were documented. In addition to the hospitalized AECOPD cohort, 110 stable COPD patients (different than the patients in the AECOPD cohort) were recruited from the St. Paul’s Hospital COPD clinic and they served as non-exacerbating COPD controls. The cohort was split into 1) a discovery set consisting of 421 AECOPD and 76 stable COPD patients and 2) a validation set consisting of 47 AECOPD and 34 stable COPD patients. The discovery set contained patients recruited between July 2012 and early April 2015, whereas, the validation set included patients recruited between late April 2015 to May 2016. Demographic data collected included age, sex, body mass index (BMI), ethnicity, current smoking status, and smoking duration. This gave an approximate ratio of 85:15 respectively. The study is registered on the publicly available ClinicalTrials.gov website with Identifier: NCT02050022 (registered January 28, 2014). The study was approved by the University of British Columbia Clinical Research Ethics Board (certificate number H11-00786). Written informed consent was provided by each participant in accordance with the Ethics Board.

**Specimens and measurement technique**

Following informed consent, blood samples were collected from patients in PAXgene^®^, EDTA, and serum tubes on day 1 and 3 of hospitalization, at discharge, and as well as on day 30 and day 90 post-admission date (see Fig 1). Blood components were processed as per standardized protocol, and stored in barcode-labelled aliquot tubes at -80°C until analysis.

Serum CRP was measured via a high-sensitivity assay on the Advia^®^ 1800 Chemistry System analyzer (Siemens Healthcare GmbH, Erlangen, Germany), in the Clinical Laboratory of St. Paul’s Hospital (Department of Pathology and Laboratory Medicine, Vancouver, BC, Canada) following standard operating procedures.

NT-proBNP was measured from EDTA whole blood specimens on the RAMP^®^ 200 (Response Biomedical Corp, Vancouver, BC, Canada). The RAMP assay is based on the principle of quantitative immunochromatography, with a measurement range of 18 to 35,000 ng/L. D-Dimer and cardiac troponin I, biomarkers of pulmonary embolism and acute myocardial infarction, which are both mimickers of infection-induced AECOPD, were measured from EDTA whole blood on the RAMP^®^ 2000, with a measurement range of 100 to 500 µg/L FEU and 0.10 to 32 µg/L, respectively.

Baseline lung function measurements were performed at the time of convalescence (i.e. at day 30 or day 90) for AECOPD patients, and at COPD clinic visits for stable COPD control patients. Spirometry was used to obtain lung function parameters after bronchodilator administration. The severity of pulmonary edema was assessed on chest X-ray images by an experienced chest radiologist, via the following criteria: none, mild (cephalization of blood flow), moderate (septal thickening), and severe (septal thickening and alveolar edema). Additional findings of possible cardiac dysfunction including pleural effusion (present/absent) and enlargement of the cardiopericardial silhouette were also recorded. The radiologist was blinded to the characteristics of the study participants.

**Statistical analysis**

The study population consisted of an AECOPD group with follow-up convalescent samples, and a stable COPD control group as shown in Table 1. Continuous variables that were not normally distributed were natural-logarithmically transformed prior to a Student’s t-test analysis. Categorical dichotomous variables are displayed as counts and were compared using a chi-square test. P-values of less than 0.05 (on a two-tailed test) were considered statistically significant. Analysis of variance (ANOVA) was performed for the time course analysis of the AECOPD patients, followed by Bonferroni’s multiple comparison post-hoc analysis for CRP and NT-proBNP. A t-test was also used to determine significant changes in the two biomarkers at each time point versus the control group, with p-values adjusted by the Bonferroni method. The associations between CRP and NT-proBNP concentrations during exacerbation onset were determined by Pearson’s correlation.

The multiple linear regression analysis was used to model the length of hospitalization (natural-log transformed) versus the two biomarkers, and was adjusted for age, sex and current smoking status. The concentrations used in this portion were from the first onset samples (i.e. day 1 or 3) for the hospitalized patients. As full follow-up has not been completed in the entire cohort, only 222 patients from the discovery set were used for this analysis.

Cox proportional hazards model was used for either CRP or NT-proBNP at exacerbation onset to predict the risk of death. 495 unique patients with mortality data were used for this portion of the analysis, and the concentrations were log2-transformed prior to analysis. Likelihood ratio test is used in calculating p-value.

Receiver-operating characteristic (ROC) curves were generated based on logistic regression models for diagnosing AECOPD (day 1) from convalescent (day 30 or 90) and stable COPD samples. We compared the area under the ROC curve (AUC) of 3 models: 1) NT-proBNP alone, 2) CRP alone, and 3) a logistic regression model built on both CRP and NT-proBNP, with discovery probabilities generated using leave-one-out cross-validation (LOOCV). The AUCs, along with the 95% confidence intervals, were compared using Hanley and McNeil method [[1](#_ENREF_1)]. In the LOOCV model, the algorithm cycled through the complete dataset, and a sample was excluded systematically (total n – 1) with each iteration. The logistic regression models were subsequently computed based on the total n – 1 samples, and the model was tested on the one sample that was left out. This method provided a way of estimating performance and minimized over-fitting of data to the discovery set as previously recommended [[2](#_ENREF_2)]. We also evaluated these models in the validation set (47 AECOPD and 34 stable COPD patients). The “optimal” cut-off value for the biomarkers was determined based on models that gave 90% specificity. Sensitivity is calculated as the number of true-positive results divided by the number of true-positive plus false-negative results. Specificity is calculated as the number of true-negative results divided by the number of true-negative plus false-positive results. All statistical analysis was performed using R.

**Supporting Information Table**

**Table A. Patient characteristics of the validation set**

|  |  | AECOPD | COPD Stable Controls | P-value |
| --- | --- | --- | --- | --- |
|  |  |  |  |  |
| Age (years) |  | 66.2±11 | 64.1±6.6 | 0.626 |
| Sex (Male %) |  | 60.0% | 66.7% | 1.000 |
| BMI (kg/m^2^) |  | 25.3±9.3 | 28.1±4.4 | 0.621 |
| Ethnicity (Caucasian %) |  | 80.0% | 100% | 0.474 |
| Smoking | Current (%) | 66.7% | 50% | 1.000 |
|  | Former (%) | 33.3% | 50% |  |
|  | Unknown (%) | 0% | 0% |  |
|  | Never (%) | 87.5% | 89.1% |  |
| Smoking Duration (pack-years) |  | 56±28.2 | 66.8±36.9 | 0.604 |
| FEV1 % predicted |  | 59.0±22.6 | 62.0±24.3 | 0.849 |
| FVC % predicted |  | 76.3±10.3 | 84.0±18.2 | 0.466 |
| FEV1 / FVC (%) |  | 57.9±15.9 | 56.9±20.1 | 0.935 |
| GOLD Stages | I | 25% | 33.3% | 1.000 |
|  | II | 50% | 33.3% |  |
|  | III | 25% | 16.7% |  |
|  | IV | 0% | 16.7% |  |
| Congestive Heart Failure |  | 0% | 16.7% | 1.000 |
| Coronary Artery Disease |  | 33.3% | 33.3% | 1.000 |
| Hypertension |  | 66.7% | 66.7% | 1.000 |
| Pulmonary Edema | None (%) | 83.3% | 69.2% | 0.684 |
|  | Mild (%) | 5.6% | 15.4% |  |
|  | Moderate-Severe (%) | 11.1% | 15.4% |  |
| D-Dimer (μg/L FEU) |  | 1135[862-1437] | 680[408-1332] | 0.163 |

Continuous variables are presented as median [IQR]. Comparisons made via t-test after natural-log transformation. Dichotomous variable are presented as counts (% total). Comparisons are made via chi-square test. D-Dimer concentrations measured from AECOPD day 1 samples. Abbreviations: FEU = Fibrinogen equivalent unit, IQR = interquartile range, and SD = standard deviation.

**References**

1. Hanley JA, McNeil BJ. The meaning and use of the area under a receiver operating characteristic (ROC) curve. Radiology. 1982;143(1):29-36.

2. Sin DD, Hollander Z, DeMarco ML, McManus BM, Ng RT. Biomarker Development for Chronic Obstructive Pulmonary Disease. From Discovery to Clinical Implementation. Am J Respir Crit Care Med. 2015;192(10):1162-1170.
